# Supplementary material for: Plant Hormone and Inorganic Ion Concentrations in the Xylem Exudate of Grafted Plants Depend on the Scion–Rootstock Combination
Source: Plants (Basel). 2022 Oct 1;11(19):2594. doi: 10.3390/plants11192594 (PMC9571263; doi:10.3390/plants11192594)
Supplement: Supplementary file 1 [file plants-11-02594-s001.zip › Figure_S1.pptx]

## Slide 1
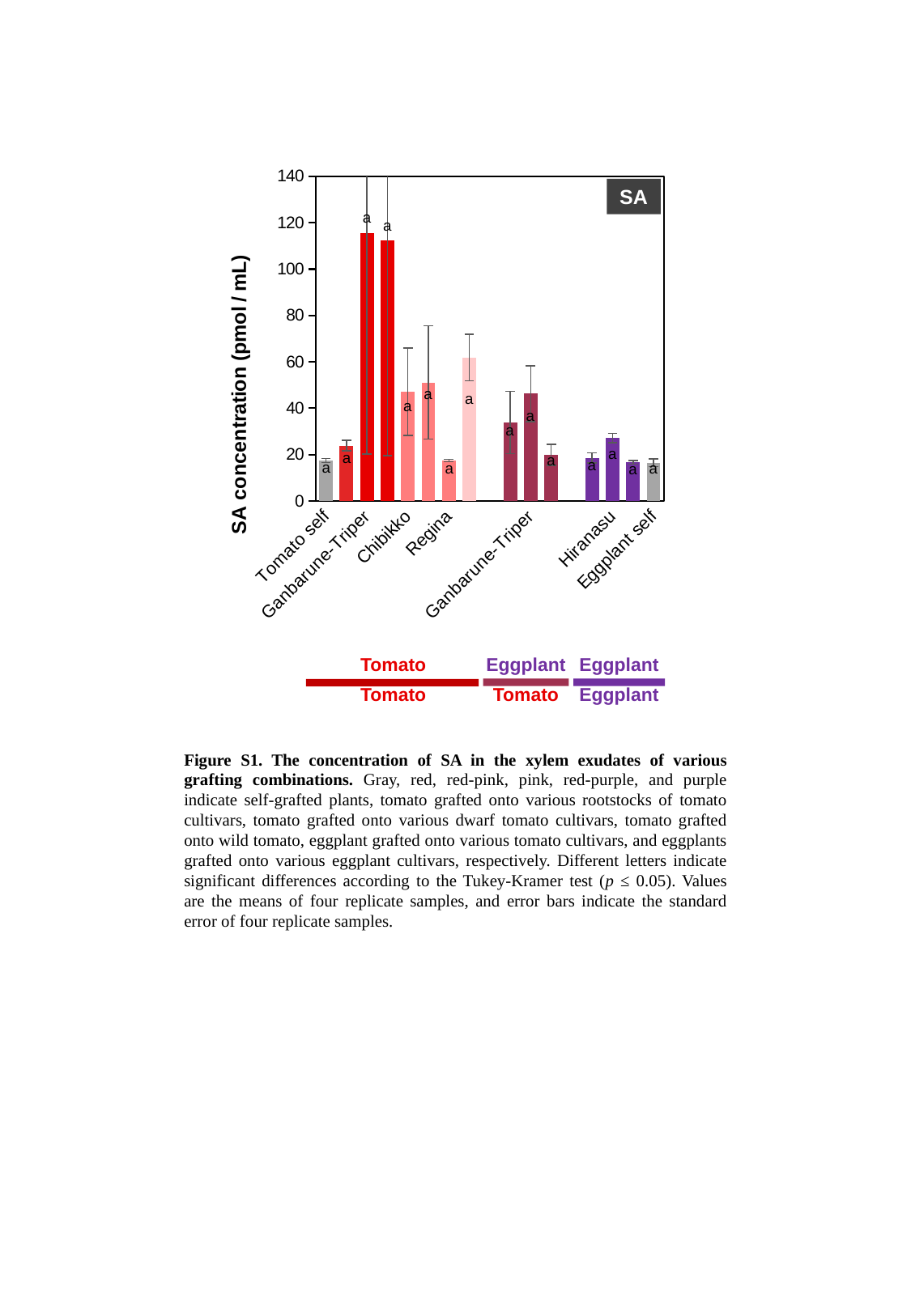

### Chart
| Category | |
|---|---|
| Tomato self | 17.490142067478182 |
| Ganbarune | 23.887784658881422 |
| Ganbarune-Triper | 115.50743513704133 |
| Spike | 112.24542884135721 |
| Chibikko | 47.097983508621994 |
| Micro-Tom | 51.09748741097717 |
| Regina | 17.464933224045723 |
| S.pennellii | 61.868939241088185 |
| | None |
| Ganbarune | 33.82344293368053 |
| Ganbarune-Triper | 46.277330203746075 |
| Spike | 19.901028934506403 |
| | None |
| Daitaro | 18.576824185266684 |
| Hiranasu | 27.10991343614205 |
| Tonashim | 16.66463110882458 |
| Eggplant self | 16.520968771602167 |SA
a
a
a
a
a
a
a
a
a
a
a
a
a
a
a
Tomato
Eggplant
Eggplant
Tomato
Tomato
Eggplant
Figure S1. The concentration of SA in the xylem exudates of various grafting combinations. Gray, red, red-pink, pink, red-purple, and purple indicate self-grafted plants, tomato grafted onto various rootstocks of tomato cultivars, tomato grafted onto various dwarf tomato cultivars, tomato grafted onto wild tomato, eggplant grafted onto various tomato cultivars, and eggplants grafted onto various eggplant cultivars, respectively. Different letters indicate significant differences according to the Tukey-Kramer test (p ≤ 0.05). Values are the means of four replicate samples, and error bars indicate the standard error of four replicate samples.
